# Effects of Anesthesia Neurotoxicity on Oligodendrocyte Development and Myelination: An In Vitro Study Using Primary Cultures

**DOI:** 10.3390/ijms27177817

**Published:** 2026-08-31

**Authors:** Qun Li, Fengying Li, Cyrus David Mintz

**Affiliations:** 1 Department of Anesthesiology, University of Maryland School of Medicine, Baltimore, MD 21201, USA; 2 Department of Anesthesiology and Critical Care Medicine, Johns Hopkins University School of Medicine, Baltimore, MD 21205, USA; fli41@jhmi.edu

**Keywords:** anesthesia neurotoxicity, isoflurane, primary culture, oligodendrocyte, oligodendrocyte progenitor cell, myelination, immunocytochemistry

## Abstract

General anesthesia allows safe and comfortable performance of surgical operations, but early-life general anesthetic (GA) exposure may cause harm to the developing brain. The mechanism for this phenomenon remains unclear. Laboratory models demonstrate a variety of effects of GA on the immature brain, but consequences of early-life anesthetic exposure on oligodendrocyte development have seldomly been reported. To better understand the potentially toxic effects of GA on brain development, this study uses oligodendrocyte progenitor cells (OPCs) from primary cultures to investigate the effects of isoflurane (ISO), a canonical inhaled GA, on oligodendrocyte development and myelination. OPCs from cerebral cortices of newborn mice (P3) were cultured and purified with the anti-O4 microbeads technique. More than 92% of cells were confirmed as OPCs. On day of in vitro (DIV) 4, OPCs were exposed to 1.5% ISO in carrier gas (5% CO_2_ and 95% air) for 4 h, or to carrier gas only as a control (CON). Immunocytochemistry (ICC) was conducted to evaluate the alteration of OPC development. By DIV 8, compared to CON group, the number of apoptotic OPCs in ISO exposure increased and the number of proliferating OPCs decreased. By DIV 12, the ratio of mature oligodendrocytes to OPCs was reduced, which indicated that oligodendrocyte differentiation was impaired. By DIV 14, the area of oligodendrocyte processes, which represents oligodendrocyte maturation, was smaller in ISO than CON. In OPC/neuron co-cultures, ISO exposure reduced the sum length of oligodendrocyte processes wrapping the neuronal axon. In conclusion, early GA exposure substantially disrupts oligodendrocyte development and myelination, which probably contributes to cognitive disorders.

## 1. Introduction

Modern general anesthesia allows for the safe and comfortable performance of several hundred million surgical procedures annually [1]. However, there is growing concern that some vulnerable categories of patients, particularly young children and individuals with underlying brain disorders, may be at risk of lasting cognitive dysfunction due to the harmful effects of anesthetics on the developing brain [2,3,4]. While the significance of developmental anesthetic neurotoxicity in children remains unclear, the predominance of animal studies has shown that exposure to general anesthetic (GA) in early development causes impaired neurocognitive performance [5,6,7,8], and that the peak period of behavioral and cognitive vulnerability to general anesthetics in rodents occurs in early postnatal life [9,10,11]. Based on these studies, the U.S. Food and Drug Administration issued a warning that lengthy or repeated exposure to general anesthetics and sedative drugs from the third trimester of prenatal development through to the first 3 years of life may cause lasting impairment in cognitive function [12]. The molecular and cellular mechanisms underlying this phenomenon remain poorly understood.

To date, most studies on anesthesia neurotoxicity have focused primarily on neuronal injury [7,13]. However, normal brain maturation depends on coordinated neuronal development and myelin formation, which is a critical developmental process in the central nervous system (CNS) that enables rapid saltatory conduction, maintains axonal integrity, and supports higher cognitive functions. Myelin is produced by oligodendrocytes, which arise from oligodendrocyte progenitor cells (OPCs) through a highly regulated process involving proliferation, migration, differentiation, and maturation [14,15]. Of note, myelination is quite incomplete in prenatal development and continues throughout childhood, rendering it a potentially vulnerable process for toxicity due to early-life GA exposure. Disruption of oligodendrocyte development during critical periods of brain maturation may result in long-lasting deficits in neural connectivity and neurological function [16,17]. Studies in neonatal and fetal non-human primates have shown that prolonged GA exposure induces significant apoptosis of oligodendrocytes throughout the developing white matter [18,19]. Furthermore, oligodendrocyte disorder during early development may compromise myelin formation and contribute to persistent neurobehavioral abnormalities observed after anesthetic exposure [20,21]. Experimental and clinical evidence has increasingly indicated that white matter alterations may represent an important component of developmental anesthetic neurotoxicity.

Although previous in vivo studies have provided valuable insights, interpretation of their findings is complicated by the systemic physiological effects of anesthesia, including alterations in cerebral blood flow, oxygenation, metabolism, and inflammatory signaling. Consequently, it remains unclear whether anesthetics exert direct toxic effects on OPCs and developing oligodendrocytes or whether the observed abnormalities arise secondarily from changes in the neural microenvironment. Therefore, an in vitro model using purified primary OPC cultures provides a valuable approach to examine the direct cellular effects of anesthetic on oligodendrocyte lineage progression and myelin formation.

**Figure 1:**
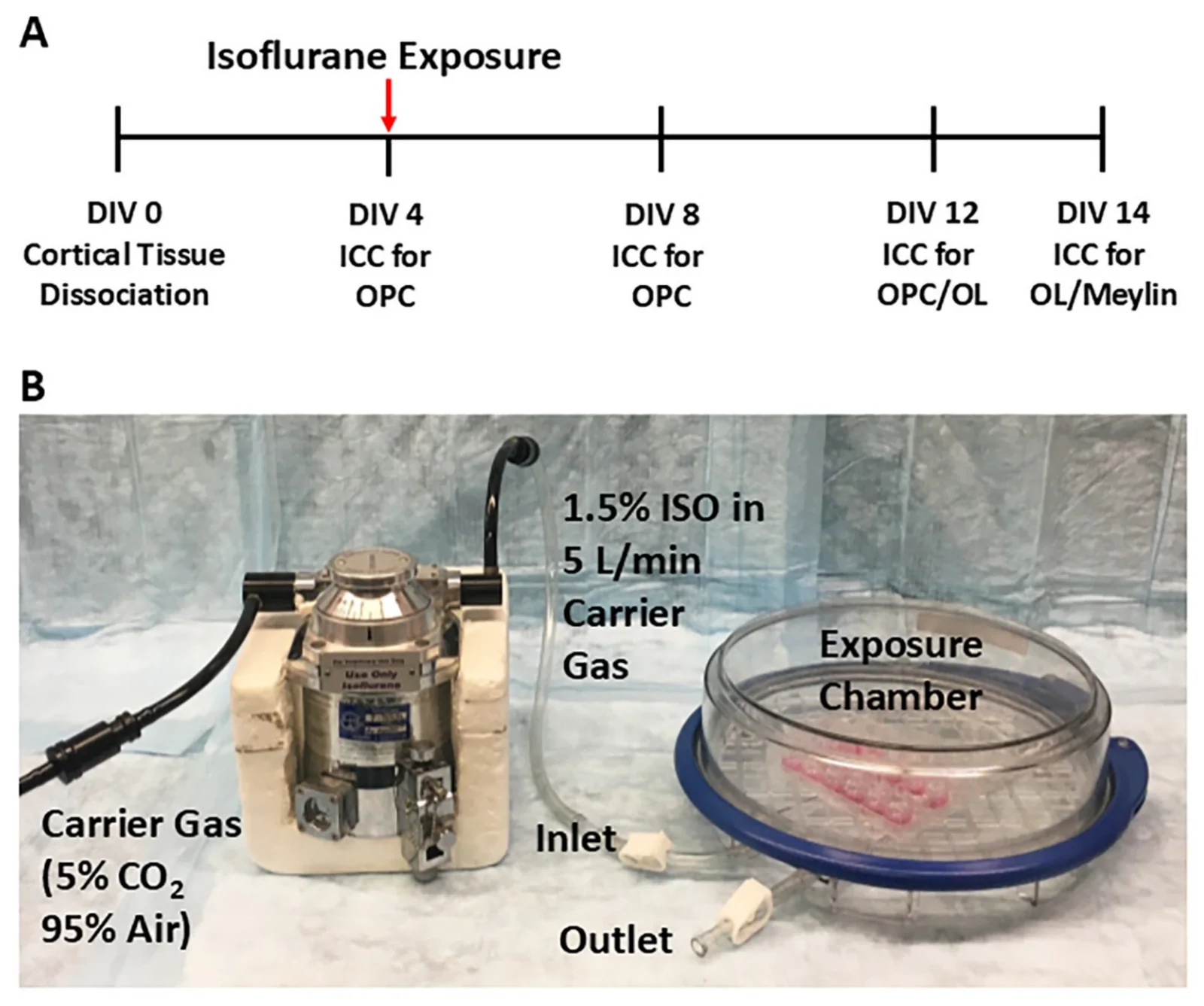
Experimental model system. (**A**) Timeline for primary culture and oligodendrocyte development. Oligodendrocyte progenitor cells (OPCs) were gained from cortical dissociation of P3 neonatal mice at DIV 0. The cultured cells were exposed to isoflurane (ISO) at DIV 4 and underwent immunocytochemistry (ICC) or TUNEL/EdU staining at different time-points. (**B**) ISO exposure paradigm. The cultured OPCs were placed in an air-tight plastic chamber, which was connected to an anesthetic vaporizer that delivers 1.5% isoflurane mixed with a carrier gas (5% CO_2_ and 95% air) for 15 min equilibration. Cultures exposed to the carrier gas alone were used as a control (CON). Then, the chamber was closed, and cultures were placed in culture incubator at 37 °C for 4 h. After ISO exposure, plates with OPCs were removed from the chamber and continuously cultured until the time-points for ICC staining.

In the present study, we employed primary cultures of OPCs derived from neonatal mouse cortex [22,23] to investigate the effects of isoflurane—one of the widely used volatile anesthetic agents in pediatric anesthesia due to its rapid onset, controllable depth of anesthesia, and favorable safety profile—on oligodendrocyte lineage development and myelination. Isoflurane was administered on the fourth day in vitro (DIV 4), a developmental stage characterized by active OPC proliferation and differentiation (Figure 1). We examined multiple aspects of oligodendrocyte development, including apoptosis, proliferation, differentiation, maturation, and myelin formation. The isoflurane exposure paradigm in this study is similar to one we applied in our previous published work, in which we investigated the effect of isoflurane exposure on oligodendrocyte development and myelination in the mouse hippocampus [21]. In addition, this concentration corresponds to that used in clinics, and its low Bispectral Index (BIS) has been recorded in pediatric surgery [24]. We hypothesized that isoflurane exposure directly disrupts OPC development and impairs subsequent myelination. Understanding the effects of anesthetics on oligodendrocyte lineage cells may provide important mechanistic insights into developmental anesthesia neurotoxicity and identify potential cellular targets for therapeutic intervention. The overall goals of the present study are to provide the background and rationale needed to facilitate clinical studies and determine whether early anesthesia exposure can cause increased risk of neural developmental disorders in pediatric patients, as well as to simultaneously uncover practical strategies to prevent this putative harmful effect of anesthetic care.

## 2. Results

### 2.1. Primary Culture Produces Purified Oligodendrocyte Progenitor Cells (OPCs)

**Figure 2:**
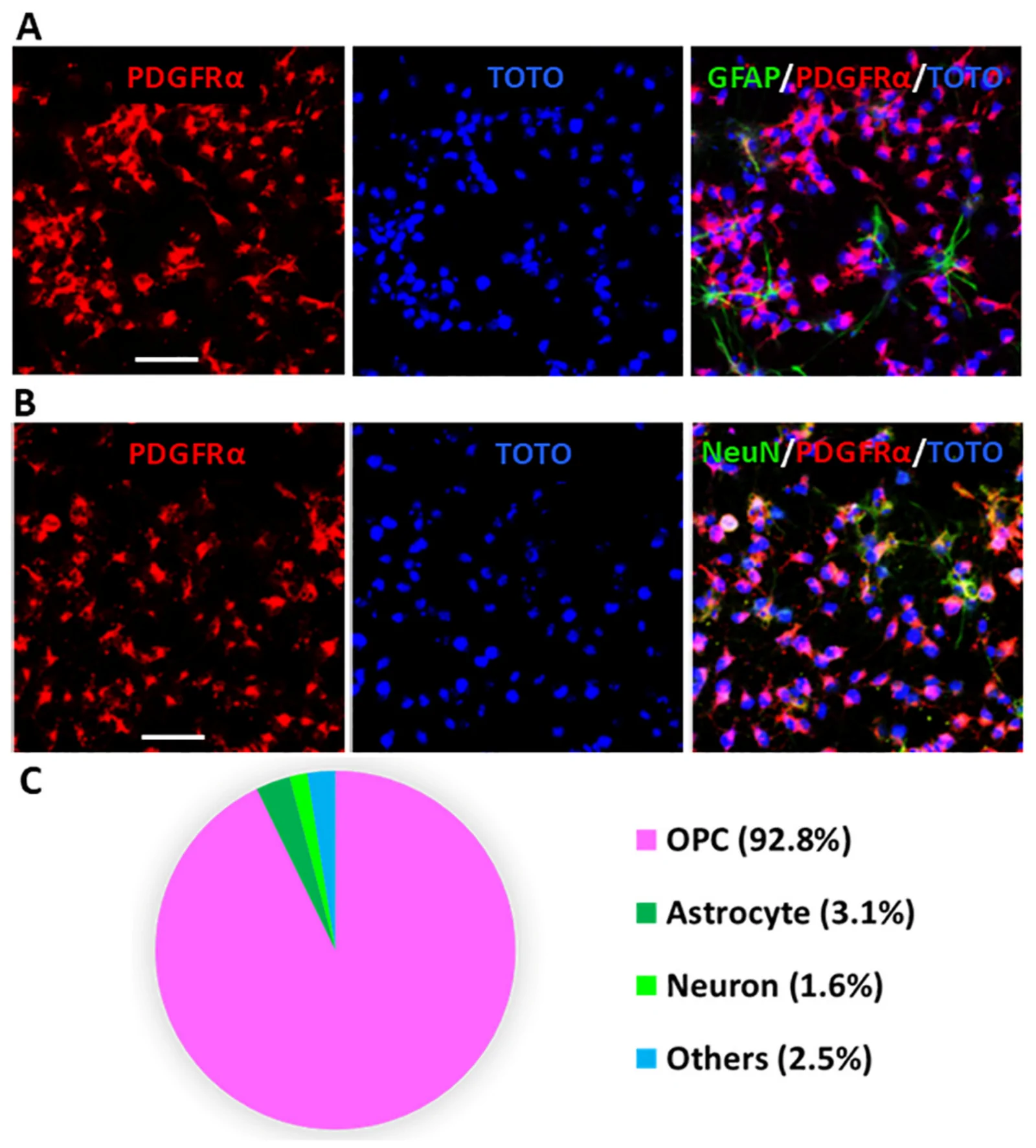
Primary cultures producing pure oligodendrocyte progenitor cells (OPCs). Cultures were processed for immunocytochemistry (ICC) on DIV 4. Platelet-derived growth factor receptor alpha (PDGFRα) labels the OPCs (red) in all cultures and TOTO stains the nuclei (blue). Pink cells are OPCs with nuclei. Glial fibrillary acidic protein (GFAP)-positive cells represent astrocytes (**A**), and neurons are labeled with neuronal nuclei (NeuN) (**B**). In total, 92.8% cells from primary cultures were observed as PDGFRα+ OPCs and very few cells as astrocytes (3.1%) and neurons (1.6%). Bars = 50 µm. (**C**) The pie chart shows the quantitative results.

First, we investigated whether the primary cultures we used in this study produce purified OPCs. In present study, cortices of newborn C57BL/6 mice (P3) were used for primary cultures, and the microbeads with anti-O4 antibody were applied to purify the OPCs [22,23]. At DIV 4, immunocytochemistry (ICC) staining was performed. Data from each well of 24-well plates represent the replicates (“*n*” number). For each group (CON vs. ISO), “*n*” equals six from three independent cultures, two wells from each. In ICC staining, more than 92% cells of TOTO-positive nuclei were immuno-labeled with platelet-derived growth factor receptor alpha (PDGFRα), which is a specific marker for OPCs. Only 3.1% were observed as glial fibrillary acidic protein (GFAP)-positive astrocytes (Figure 2A), and 1.6% were neuronal nuclei (NeuN)-positive neurons (Figure 2B). This indicates that relatively purified OPCs can be gained from our primary cultures (Figure 2C).

### 2.2. Effect of Isoflurane Exposure on Apoptosis of OPCs

**Figure 3:**
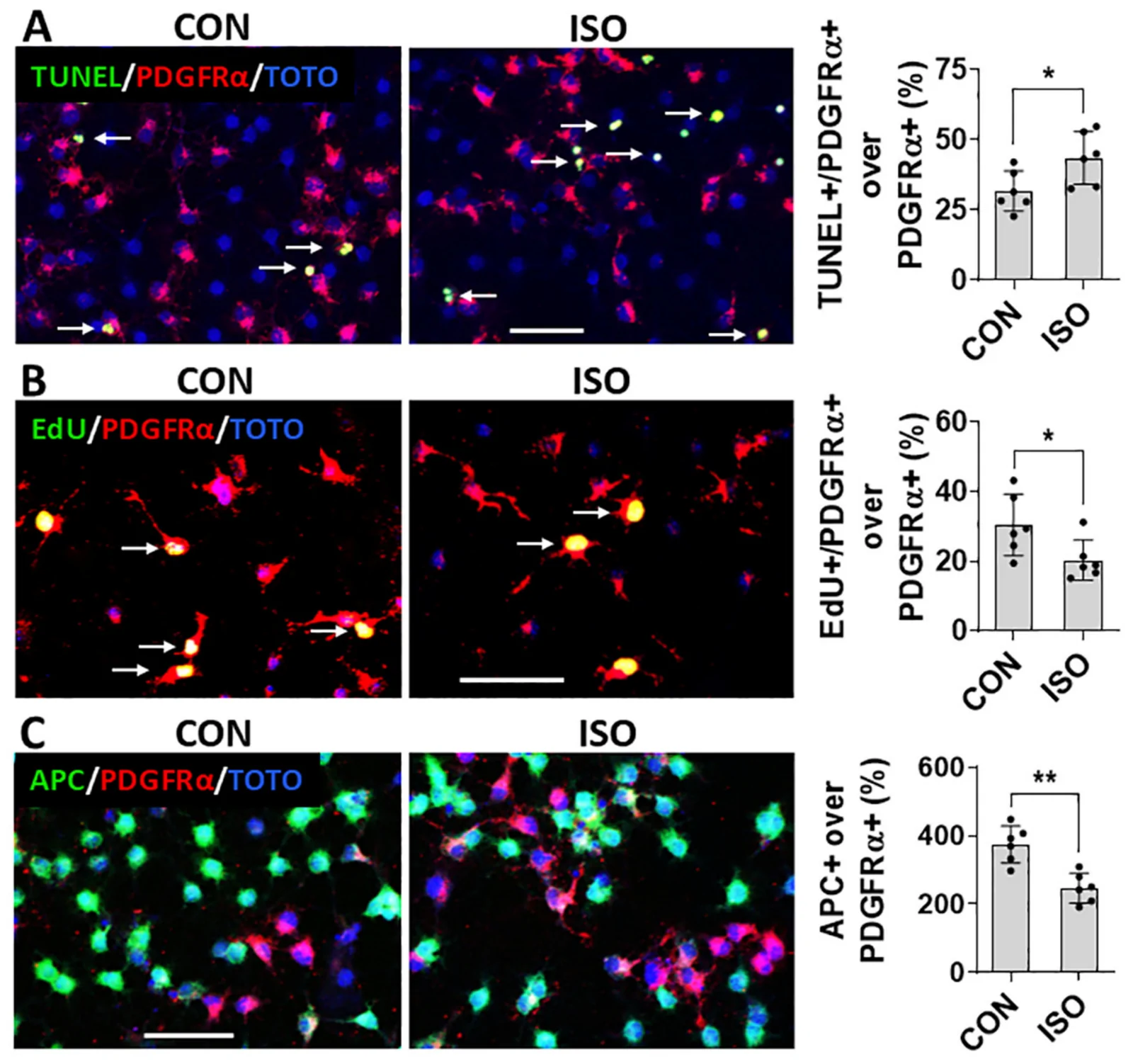
Isoflurane (ISO) exposure increases OPC apoptosis and decreases OPC proliferation and differentiation. (**A**) On DIV 8, double-labeling of TUNEL (green) and PDGFRα (red) indicates apoptosis of OPCs (yellow; arrows). ISO exposure increases the ratio of TUNEL+/PDGFRα+ over total PDGFRα+ cells than the control (CON). (**B**) EdU+/PDGFRα+ proliferating OPCs are observed (arrows, yellow). There is a significantly lower number of EdU+/PGDFRα+ cells in the ISO group than in CON. (**C**) ISO inhibits oligodendrocyte differentiation (DIV 12). APC (green) and PDGFRα (red) label mature oligodendrocytes and OPCs, respectively. Pink cells are PDGFRα+ OPCs with nuclei. The ratio of oligodendrocytes over OPCs is significantly decreased in ISO-exposed than CON cultures. Bars = 50 µm. *: *p* < 0.05; **: *p* < 0.01.

Given the extensive literature suggesting that early developmental anesthetic exposure can cause an increase in apoptosis in a range of different brain cell types [17,18,19], we calculated the percentage of apoptotic OPCs that were immuno-labeled with PDGFRα and TUNEL (terminal deoxynucleotidyl transferase dUTP nick end labeling) staining in isoflurane exposure vs. control cultures. TUNEL is a widely used technique to detect DNA fragmentation in programmed cell death (apoptosis) [25,26]. Overall, 31.8 ± 6.5% of TUNEL+/PDGFRα+ double-labeled apoptotic OPCs out of all OPCs (PDGFRα+) were observed in the control, and this number increased to 43.7 ± 8.6% in the isoflurane group (*p* < 0.05) (Figure 3A). These findings suggest that isoflurane exposure results in a substantial upregulation of apoptotic pathways in developing OPCs.

### 2.3. Effect of Isoflurane Exposure on OPC Proliferation

Our previous report on rodent animal models has shown evidence that early exposure to anesthetic can cause alterations in OPC proliferation, which in turn result in structural changes and functional impairments [21]. To evaluate OPC proliferation levels in vitro, cultured cells were immuno-labeled with an antibody against PDGFRα and stained with 5-ethynyl-2′-deoxyuridine (EdU), a specific marker for cellular division, on DIV 8 [27]. EdU was applied to OPC cultures 14 h before harvest of cells for incorporation into the newly synthesized DNA during the S-phase. EdU and PDGFRα double-labeling represents proliferating OPCs. In the control group, 30.5 ± 8.1% EdU+/PDGFRα+ cells were observed, and this number significantly decreased to 20.3 ± 5.3% with isoflurane exposure (*p* < 0.05) (Figure 3B). These data indicate that isoflurane exposure impairs proliferation of cultured OPCs.

### 2.4. Effect of Isoflurane Exposure on OPC Differentiation

We then performed double-ICC for adenomatous polyposis coli (APC), which labels mature oligodendrocytes and PDGFRα, and applied the ratio of oligodendrocytes to OPCs as a parameter to evaluate oligodendrocyte differentiation. There are numerous PDGFRα+ and APC+ cells visualized in the cultures, but almost no double-labeled cells were observed, confirming the specificity of the markers. The ratio of the number of APC-positive oligodendrocytes to the number of PDGFRα-labeled OPCs in isoflurane-exposed culture (247.1 ± 39.8%) is revealed to be significantly lower than in control conditions (374.4 ± 49.0%; *p* < 0.01) (Figure 3C). These data suggest that isoflurane exposure results in a lasting impairment of the differentiation of OPCs into the mature oligodendrocyte phenotype.

### 2.5. Effect of Isoflurane Exposure on Oligodendrocyte Maturation

**Figure 4:**
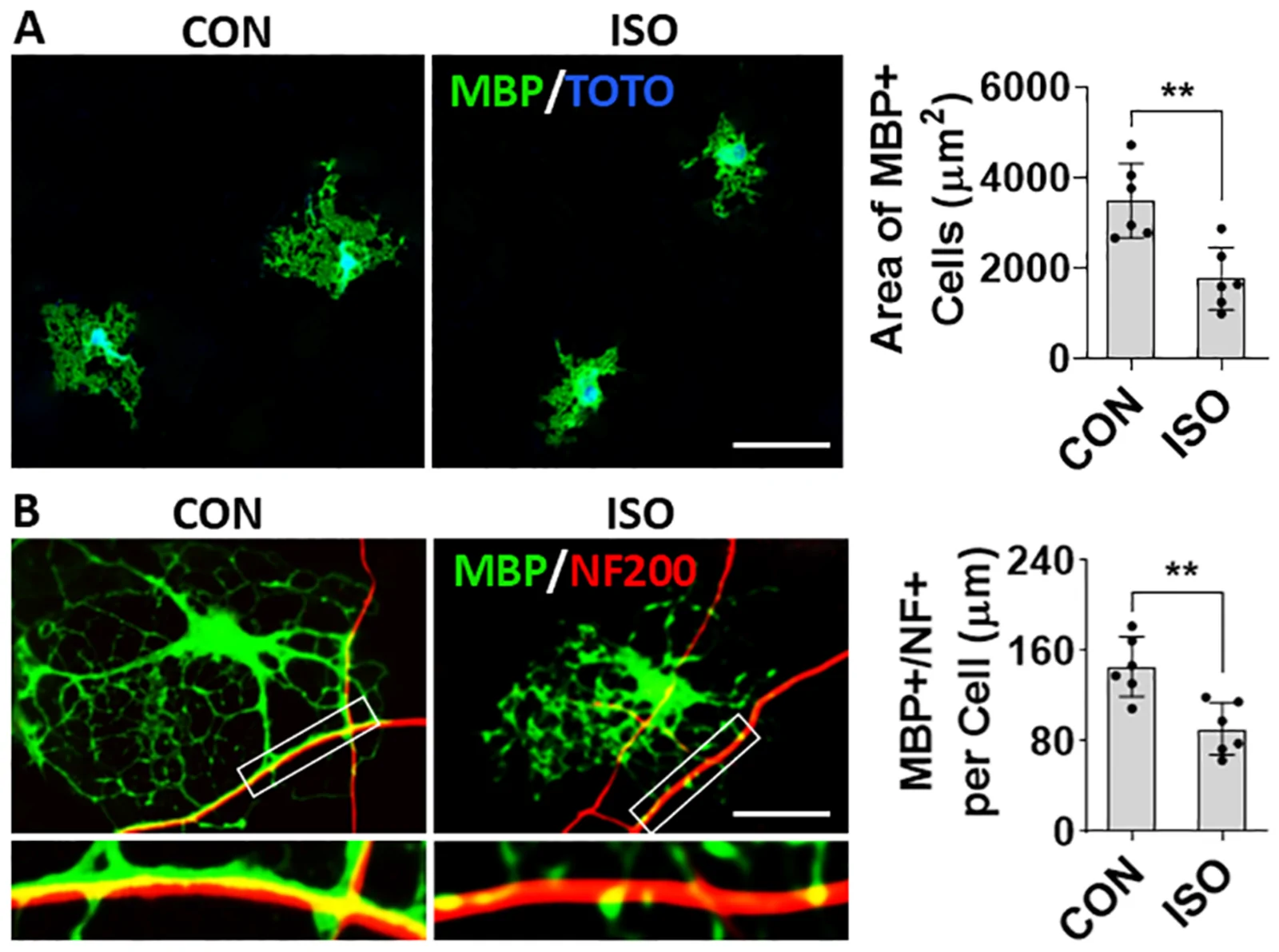
Isoflurane exposure impedes oligodendrocyte maturation and myelination. (**A**) On DIV 14, MBP staining for the processes of oligodendrocytes reveals the maturation of these cells, with TOTO labeling the nuclei; isoflurane exposure reduces the area of MBP+ oligodendrocytes processes. Bar = 50 µm. (**B**) DIV 14 of OPC/neuron co-culture. Isoflurane decreases MBP+ processes (green) wrapping the NF200+ neuronal axons (red) more than the control. Yellow represents myelination. Bar = 20 µm. The boxes in low-power photos represent the locations of high-power images below. **: *p* < 0.01.

Anesthetic exposure has also been shown to disrupt the formation of myelin through impeding the maturation of oligodendrocyte [21,28]. An important characteristic of maturation is the perfect growth of oligodendrocyte processes. To test the effects of anesthetics on oligodendrocyte maturation, cultured cells were immuno-labeled with myelin basic protein (MBP), which reveals all process arbors, and the total areas of these process arbors were quantitatively analyzed. In the control, the whole-cell area is measured at 3486.2 ± 751 µm^2^, which decreases to 1762.7 ± 630.6 µm^2^ in the isoflurane exposure group. This represents a significant decrease (*p* < 0.01) (Figure 4A).

### 2.6. Effect of Isoflurane Exposure on Myelination

Next, we investigated the effects of isoflurane exposure on myelin development. In our OPC/neuron co-culture model, myelin formation is detected by oligodendrocyte processes wrapping the neuronal axons. We measured the sum length of myelinated axons, which were identified as processes co-immuno-labeled with the axon marker neurofilament (NF) 200 and MBP. Early exposure to isoflurane significantly decreased the total length of MBP+/NF200+ oligodendrocyte process-wrapped axons (146.2 ± 24.3 µm vs. 91.2 ± 20.9 µm per OPC; *p* < 0.01) (Figure 4B). Taken together, these findings suggest that early developmental exposure to isoflurane results in a lasting reduction in myelination in vitro. This is consistent with a lasting and substantial neurotoxic effect of early developmental anesthetic exposure to rodent and human brain tissue [19,21,28].

## 3. Discussion

In the present research, we use a primary culture model to show that early exposure of OPCs to isoflurane, a frequently used general anesthetic (GA) that is a prototype for sevoflurane and desflurane and thus represents the potent inhaled agents, significantly disrupts multiple stages of oligodendrocyte development in vitro. Specifically, isoflurane increased OPC apoptosis, reduced OPC proliferation, impaired differentiation into mature oligodendrocytes, diminished oligodendrocyte process development, and decreased axonal ensheathment. These findings suggest that oligodendrocytes are important cellular targets of anesthetic neurotoxicity, and that impaired oligodendrocyte maturation may contribute to the adverse neurodevelopmental outcomes associated with early-life anesthetic exposure in patients. In our experiments, we performed primary cultures in which OPCs were derived from cortices of neonatal mice. In this culture procedure, microbeads with anti-O4 antibodies were applied to purify OPCs [22,23]. According to ICC results, we gained relatively pure OPCs, but there was still a low portion of other cell phenotypes present, such as neurons and astrocytes. We used our exposure device that was developed in our laboratory [7,29,30,31,32] to expose cultured OPCs to 1.5% isoflurane for four hours. Before the formal experiment, we tested different doses of isoflurane, including 0.5%; 1%; 1.5%; and 2%. The 1.5% isoflurane concentration chosen in this study was the lowest dose that caused significant developmental alterations in the OPC cultures. In addition, this dose corresponds to similar experiments from other researchers, in which the minimum alveolar concentration (MAC) of mice was tested, and a comparison between the water/gas partition coefficient of isoflurane in vitro and the blood/gas partition coefficient in vivo was per formed [33,34].

Most investigations in developmental anesthesia neurotoxicity have focused on disfunction of neurons [13,35]. However, brain function and development are heavily dependent on myelin-forming oligodendrocytes, which undergo critical developmental events during a putative window of vulnerability to anesthetic toxicity. Myelination is essential in establishing connectivity in the growing brain by facilitating rapid and synchronized information transfer across the nervous system. Myelin, rather than simply providing insulation to facilitate signal transduction in the developed brain, is critically important for the development of brain circuitry [36]. Myelination involves proliferation and differentiation of oligodendrocyte progenitor cells (OPCs), maturation of oligodendrocytes, and ensheathment of neuronal axons. Damage to or loss of myelin function is associated with many neurologic, psychiatric, and cognitive disorders [37].

In both in vitro and in vivo studies, including our previous study in human brain organoids [28], a certain grade of oligodendrocyte apoptosis happens especially in early development stages. In cell cultures, the apoptotic rate depends on culture conditions, such as how NGF might increase the apoptotic rate from 10% to 43% [38]. In the precent study, the percentage of apoptotic cells in both the control (31.8%) and isoflurane (ISO) groups (43.7%) was higher than some other works; the reason for which is probably due to our cell counting method. When we counted PDGFRα+ OPCs, we emphasized they should have intact outlines of cell bodies (Figure 3A); this probably led to the total OPC number being lower, while the percentage of double-labeled cells over PDGFRα+ was higher than actual. In fact, the data we showed in this study indicate that, in the same culture conditions, ISO exposure increased the ratio of apoptotic over total OPCs. In the in vivo study, it was also confirmed that OPC apoptosis was extensively observed in the development of the brain, and early ISO exposure increased the number of apoptotic OPCs [18].

The process of oligodendrocyte development and myelin formation occurs based on an intrinsic program that is modulated by neurotransmitters and electrical activity in the CNS [39,40,41]. Oligodendrocyte lineage cells express α-amino-3-hydroxy-5-methyl- 4-isoxazolepropionic acid (AMPA), kainite, and N-methyl-d-aspartate (NMDA) receptors that are excited by glutamate and mediate Na^+^ and Ca^2+^ entry. By interacting with these receptors, glutamate increases the downstream phosphorylation of the cyclic adenosine monophosphate (cAMP) response element-binding protein (CREB) and the release of calcium from intracellular stores, thereby promoting OPC survival, proliferation, differentiation, maturation, and myelination [42]. Isoflurane inhibits NMDA receptor-mediated currents and, at clinically relevant concentrations, can reduce AMPA/kainate receptor-mediated currents as well. The block is dose-dependent and tends to reduce channel open probability and conductance. So, as an agonist of γ-aminobutyric acid type A (GABA-A) and glycine receptors, [43] as well as a potential glutamatergic inhibitor, [44] isoflurane suppresses excitatory neurotransmission [45]. It raises the possibility that a profound direct action on OPCs, which express both GABA and glutamate receptors, is caused by isoflurane during a critical period in development, and that this may alter the developmental program, thus resulting in deficits in myelination.

In our previous neuron/astrocyte co-culture study, isoflurane exposure caused a reduction in the brain-derived neurotrophic factor (BDNF) in the culture medium [31]. BDNF, through the tropomyosin receptor kinase B (trkB) receptor expressed in OPCs and the mitogen-activated protein kinase (MAPK) pathway, enhances DNA synthesis, thereby promoting the differentiation of OPCs in cultures [46,47,48]. In BDNF knockout animals, the number of OPCs is selectively decreased in the forebrain. Meanwhile, the reduction in BDNF also inhibits the level of MBPs, which is a myelin-formation-associated glycoprotein [49]. These data indicate that BDNF plays an important role in OPC proliferation and differentiation and is necessary for myelin formation.

Our previous work using a rodent model has indicated that exposure to isoflurane disrupts oligodendrocyte development of the hippocampus in the early postnatal period by inappropriately increasing activity in the mammalian target of rapamycin (mTOR) pathway, and we found that both histological and behavioral changes could be reversed by pharmacologic mTOR inhibition [21]. The mTOR pathway is an intracellular signaling pathway that senses and integrates a variety of environmental cues to regulate organismal growth and homeostasis. It is involved in many major cellular processes, including proliferation, differentiation, apoptosis, metabolism, transmitter release, and other biological processes [50]. Two structurally and functionally distinct mTOR-containing complexes have been identified in oligodendrocytes. The first, mTOR complex 1 (mTORC1), contains the adaptor protein Raptor, which influences MBP expression via an alternative mechanism and is sensitive to the drug rapamycin. The second complex, mTORC2, contains Ritor, and it is thought to control myelin gene expression at the mRNA level and is relatively rapamycin insensitive [51]. The mTOR pathway itself plays a complex role in myelination, as mTOR activity can either enhance or suppress oligodendrocyte development depending on the context. A study using a mouse line with oligodendrocyte-specific knockdown of mTOR in the CNS has provided evidence that mTOR is essential for oligodendrocyte development and myelination [52]. The inhibition of mTOR via rapamycin in cultured OPCs results in oligodendrocyte deficits along with reduced expression of major myelin proteins and mRNAs [53,54,55]. In contrast, the activation of mTOR, induced by tuberous sclerosis complex-1 or -2 gene mutations in OPCs, produces white matter abnormalities, including myelin deficits in the CNS [56,57,58]. So, oligodendrocyte development and myelination may be dependent on the precise balance and timing of mTOR signaling. Either increasing or decreasing the levels of mTOR complex 1 activity interferes with oligodendrocyte differentiation and potentially causes hypomyelination [59].

## 4. Materials and Methods

### 4.1. Primary Culture for Oligodendrocyte Progenitor Cells (OPCs)

In our primary cultures, OPCs were gained from cortices of newborn C57BL/6 mice. Pups were not sexed prior to sacrifice. On postnatal day 3 (P3), pups were sprayed with 70% ethanol spray for sterilization and underwent decapitation. Under the dissection microscope, the head skin and skull were removed, and brains were immediately placed in a Petri dish containing ice-cold Hank’s Balanced Salt Solution (HBSS) without Ca^2+^ and Mg^2+^ (Thermo Fisher Scientific, Waltham, MA, USA). The cerebellum, olfactory bulbs, brain stem, and hippocampus were pinched off with fine tweezers. Meninges were peeled off from ventral side. Cortices of both hemispheres were dissected, with additional care taken to avoid including any white matter, and then minced with a razor blade. Tissue was collected in HBSS without Ca^2+^/Mg^2+^ in 15 mL centrifuge tube and centrifuged at 300× *g* for 2 min. Afterward, cortical tissue was processed using the Neural Tissue Dissociation kit (Miltenyi Biotec Inc., Auburn, CA, USA) according to the manufacturer’s instructions. HBSS was removed and a pre-warmed “Enzyme Mix 1” (100 µL Enzyme P plus 3800 µL Buffer X for three brains) was added. Tissue samples were incubated at 37 °C under continuous rotation for 15 min. “Enzyme Mix 2” (20 µL Enzyme A + 40 µL Buffer Y) was added, and tissues were incubated at 37 °C for 10 min. Tissue samples were mechanically dissociated by repeated suck/push using a 1 mL pipette with two tips of decreasing diameters (cut from 1 mL tips), 15 times for each. After 10 min incubation at 37 °C, cell suspension was applied to MACS SmartStrainers (70 µm, then 40 µm) and collected in HBSS with Ca^2+^/Mg^2+^. Centrifugation was conducted at 300× *g* for 10 min, and then the supernatant was completely aspirated. OPC purification was performed using anti-O4 isolation beads, MACS MS columns, and a MACS MiniSeparator, according to the manufacturer’s instructions (all Miltenyi Biotec). A ratio of 1:10 with anti-O4 microbeads in BSA buffer (1:20 with PBS from MACS BSA Stock Solution) were added to the cell pellet, mixed well, and then incubated at 4 °C for 15 min. Cells were washed with BSA buffer and 500 µL of cell suspension was applied onto the MACS MS column. The magnetically labeled OPCs were immediately flushed out by firmly pushing the plunger into the column. The 24-well plates were used for OPC cultures. For each well, a sterilized 12 mm diameter coverslip with a Laminin coating (Neuvitro, Camas, WA, USA) was placed on the bottom of the well so that cells were cultured on the surface of the coverslip. The total number of cells was counted by mixing one part of the cell suspension with one part of a trypan blue solution. Cells were plated at a density of 40,000 cells in 50 µL OPC culture medium per well and allowed to adhere for 30 min in a culture incubator maintained at 37 °C and 5% CO_2_. It is important to note that the cell suspension should be spread across the entire surface of the coverslips sitting in the culture wells. Then, the wells were gently flooded with the culture medium up to the total volume of 300 µL. The OPC culture medium consisted of the following: DMEM:F12 (Thermo Fisher Scientific,), N2B (1:1000 stock; Stem Cell Technologies, Vancouver, BC, Canada), SM1 (1:50; Stem Cell Technologies), penicillin/streptomycin (1:200; Thermo Fisher Scientific), N-Acetyl-Cysteine (5 mg/mL; Millipore-Sigma, Burlington, MA, USA), forskolin (5 µM; Calbiochem, San Diego, CA, USA), CNTF (10 ng/mL; Peprotech, Cranbury, NJ, USA), PDGF-AA (20 ng/mL; Peprotech, Cranbury, NJ, USA), and NT-3 (1 ng/mL; Peprotech, Cranbury, NJ, USA). To induce OPC differentiation, the day after cell plating, the OPC culture medium was replaced with a medium no longer containing PDGF-AA and NT3 but supplemented with T3 (4 ng/mL; Millipore-Sigma, Burlington, MA, USA). Cells were then continually cultured, and the medium was changed every 2 days [22,23].

### 4.2. OPC and Neuron Co-Culture

For the myelination study, an OPC and neuron co-culture was conducted. The dissection of P3 mouse cortices was similar with that described in 4.1. Briefly, brains were collected in HBSS without Ca^2+^/Mg^2+^. After carefully removing the meninges, the cortical tissue was cut into small pieces using a razor blade. The minced tissue was then transferred into a 15 mL centrifuge tube with 1 mL of Trypsin-EDTA (Sigma, Indianapolis, IN, USA) and incubated for 15 min at 37 °C. The enzymatic reaction was stopped by mixing the tissue with 1.5 mL of trypsin inhibitor DNase-I solution (0.05% soybean trypsin inhibitor, 0.02% DNase-I, and 0.3% BSA in DMEM), and the tissue suspension was then centrifuged at 300× *g* for 5 min. The supernatant was replaced with 5 mL of the plating medium (50% normal horse serum and 20% HBSS with Ca^2+^/Mg^2+^ in DMEM). Tissues were then titrated with a 1 mL pipette tip 15 times. The dissociated cell suspension was then passed through 70 μm and 40 μm cell strainers. After the cell counting, 50 µL of the suspension was seeded into each well of the 24-well plates (12 mm diameter coverslips coated with Laminin sitting on bottom of wells) at a density of 40,000. After 30 min for adhesion, 250 μL of the myelination medium was slowly added into each well. The myelination medium was a 1:1 combination of N2 medium and neural basal medium (NBM). The final concentrations of the individual components in the N2 medium (DMEM-F12-based, high-glucose, Invitrogen) are listed as follows: insulin (10 μg/mL), NGF (50 ng/mL), NT-3 (10 ng/mL), transferrin (50 μg/mL), sodium selenite (5.2 ng/mL), hydrocortisone (18 ng/mL), putrescine (16 μg/mL), progesterone (6.3 ng/mL), biotin (10 ng/mL), N-acetyl-L-cysteine (5 μg/mL), BSA (0.1%), and penicillin–streptomycin (50 units/mL). The medium was changed every 2 days. From DIV 8, insulin, NGF, and NT-3 were excluded while T3 was introduced into the N2 medium. The ratio of insulin-free N2 to NBM was adjusted to 4:1 to prevent neuron overgrowth [60].

### 4.3. Isoflurane Exposure

On DIV 4, isoflurane exposure to OPCs was performed in a sterile environment. Briefly, 24-well plates with cultures were removed from the incubator (5% CO_2_, 37 °C), placed in a fume hood, and randomly divided into CON and ISO groups. The plates were placed in identical air-tight, humidified chambers (Billups-Rothenberg, Del Mar, CA, USA), as previously described [27,28,29]. Before the exposure day, chambers were thoroughly cleaned with a 70% ethanol spray and were UV-irradiated overnight. After the plates were placed on the mesh of the chamber (lids were placed beside), the chambers were sealed. Both the gas inlet and outlet remained open at this time. The chambers for the isoflurane exposure group were connected to an agent-specific tabletop portable anesthetic vaporizer (Supera-Vet, Vaporizer Sales and Services Inc., Rockmart, GA, USA), which delivered 1.5% isoflurane mixed with a carrier gas (5% CO_2_ and 95% air). A calibrated flowmeter was used to deliver the carrier gas at a flow rate of 5 L/min. Cultures in the control group were exposed to the carrier gas alone. After a 15 min equilibration, the gas inlet and outlet of the chamber were tightly clipped, and the chambers with cultured OPCs were placed in the incubator to maintain the temperature at 37 °C (Figure 1B). After 4 h of exposure, the chambers were removed from the incubator. The chambers were unsealed, and the isoflurane mix was released. The cultures were placed back in the incubator with the medium being changed once every other day.

### 4.4. Immunocytochemistry (ICC)

At the selected time-points (Figure 1A), cultured cells were harvested for ICC. Cultured cells were washed three times with 0.1 M phosphate-buffered saline (PBS) and then fixed with 4% paraformaldehyde (PFA) in PBS for 30 min. After 3 × 10 min washing with PBS again, cells were permeabilized in PBS with 0.1% Triton (PBST) at 4 °C for 30 min and blocked in 10% normal goat serum (NGS) for 60 min. The cultured cells were incubated with the following primary antibodies in 10% NGS in PBS overnight at 4 °C: (1) mouse anti-platelet-derived growth factor receptor alpha (PDGFRα, 1:200; EMD Millipore, Temecula, CA, USA) for OPCs mixed with rabbit anti-glial fibrillary acidic protein (GFAP) for astrocytes (1:400; Millipore Sigma, Burlington, MA, USA); (2) mouse anti-PDGFRα mixed with rabbit anti-neuronal nuclei (NeuN) (1:300; EDM Millipore, USA) for staining of OPCs and neurons; (3) mouse anti-PDGFRα after TUNEL staining for OPC apoptosis; (4) mouse anti-PDGFRα after EdU staining for OPC proliferation; (5) mouse anti-PDGFRα mixed with rabbit anti-adenomatous polyposis coli-CC1 (APC-CC1) (1:300; Millipore-Sigma, Burlington, MA, USA) for oligodendrocyte differentiation; (6) mouse anti-myelin basic protein (MBP) for the process arbor of mature oligodendrocytes (1:500; Santa Cruz Biotechnology, Dallas, TX, USA); and (7) mouse anti-MBP mixed with rabbit anti-neurofilament 200 (NF200) (1:100; Sigma-Aldrich, St. Louis, MO, USA) for myelination of axon. After 3 × 10 min washes with PBS, cultures were incubated in the following secondary antibodies in 3% NGS for 3 h at room temperature: Alexa 488-conjugated goat anti-rabbit IgG (1:600; Invitrogen, Carlsbad, CA, USA) mixed with Cy3-conjugated goat anti-mouse IgG (1:1000; Jackson ImmunoResearch Labs, West Grove, PA, USA) or Alexa 488–goat anti-mouse IgG (1:600; Invitrogen) mixed with Cy3-conjugated goat anti-rabbit IgG (1:1000; Jackson ImmunoResearch Labs, West Grove, PA, USA). A ratio of 1:10,000 TOTO was added in secondary antibodies to label the nuclei. After 3 × 10 min washing with PBS, laminin-coated coverslips, with cells cultured on the bottom of the wells, were moved out, air-dried, turned over, and mounted on glass slides with ProLong antifade reagent (Themo Fisher Scientific, Eugene, OR, USA) [21,28,29].

### 4.5. TUNEL Staining

TUNEL staining was performed for the detection of cell apoptosis. On DIV 8, selected OPC cultures were fixed with 4% PFA as described in Section 4.4. After 3 × 10 min washing with PBS, cells were permeabilized in PBS with 0.1% Triton (PBST) at 4 °C for 30 min. TUNEL reactions were conducted by using the In Situ Cell Death Detection Kit (Cat#: 11684795910, Sigma-Aldrich, St. Louis, MO, USA). First, 50 µL of the Enzyme Solution (containing terminal deoxynucleotidyl transferase; Vial 1) was added to 450 µL of the Label Solution (nucleotide mixture in reaction buffer; Vial 2) to obtain a 500 µL TUNEL Reaction Mixture. Afterward, the reaction mixture was added to OPC cultures (250 µL for each well), which were incubated for 60 min at 37 °C in a humidified atmosphere in the dark. After 3 × 10 min washing, the cultures were immediately processed with further ICC procedures. TUNEL-positive cells are identified by green fluorescence labeling (488 nm channel) in the nuclei, and TUNEL/PDGFRα double-stained OPCs appear as a yellow color (Figure 3A) [25,26].

### 4.6. EdU Staining

To detect OPC proliferation, 5-ethynyl-2′-deoxyuridine (EdU) staining was conducted with a Click-iT Plus EdU Imaging Kit (Molecular Probes, Carlsbad, CA, USA). EdU from the kit was incorporated into DNA during cell division and the detection was based on a click reaction. On DIV 7, 1 µM EdU was added to the culture medium for 16 h, and cells were fixed with 4% PFA the next day. After 3 × 10 min washing with PBS and 3% BSA, cultures were permeabilized in 0.5% triton in PBS for 20 min. Following another 3 × 10 min washes, cells were incubated in a reaction cocktail, which included (i) 430 µL of a reaction buffer; (ii) 20 µL of a copper protectant; (iii) 1.2 µL of Alexa 488; and (iv) 50 µL of the EdU buffer additive, for 30 min in dark [27]. Following 3× washing with PBS, ICC for PDGFRα was processed.

### 4.7. Quantitative Analysis of ICC, TUNEL, and EdU Labeling

The ICC-labeled (or double-labeled with TUNEL/EdU) cells on coverslips were observed and their photos taken at 10× or 20× magnification using a Leica 4000 confocal microscope (Leica, Wetzlar, Germany). The profiles of cell bodies were identified with green (Alexa 488) or red (Cy3) channels, and all nuclei were stained with TOTO (blue). In each staining, six coverslips from each group (CON or ISO) with immuno-labeled cells were randomly selected from three independent batches of cultures, with 2 from each for further quantitative analysis (*n* = 6). For each coverslip, images from five fields (up, down, left, right, center) were randomly selected. The average numbers of five fields were calculated. Identical photo exposure was set for both isoflurane exposure and control groups. Based on the photos taken from these images, data analysis was quantitatively conducted with ImageJ (NIH, 1.52a) [21,28]. For all analysis, two researchers participated in the experiments. The experimenter performing image analysis was blinded to the experimental grouping in all cases.

#### 4.7.1. Cell Counting

In ICC-staining cases, PDGFRα-, GFAP-, NeuN-, APC-, PDGFRα/TUNEL-, and PDGFRα/EdUEdu-labeled cells were counted. The criteria for TUNEL- and EdU-positive cells were labeling nuclei as dead or dividing cells. For ICC labeling, immunoreactivity was seen in cytoplasm and processes, and the profiles of cells were identified. In the ImageJ program, low magnification images (10×) were opened and initialized. First, the “Plugins,” “Analysis,” and “Cell Counter” tools were selected, and each labeled cell inside the image was clicked, through which each counted cell was marked, preventing the same cell from being counted twice. The number of counted cells in each image was automatically recorded after selecting the “Analyze” and “Measure” tools. The number of labeled cells in each image was calculated. We then used the ratio of PDGFRα-positive cells to TOTO-positive cells, the ratio of GFAP+ to TOTO+ cells, and the ratio of NeuN+ to TOTO+ cells to evaluate the percentage of each phenotype and estimate the purification of the OPCs (Figure 2). Also, the ratios of TUNEL+ or EdU+ OPCs to the total PDGFRα+ OPCs, as well as APC-positive cells to PDGFRα+, were calculated to determine OPC apoptosis, OPC proliferation, and differentiation of oligodendrocyte lineage cells (Figure 3) [21,28].

#### 4.7.2. Measurement of the Area of Mature Oligodendrocytes

For the study of oligodendrocyte maturation, higher magnification (20×) images of MBP+ cells were analyzed by measuring the area of oligodendrocytes with ImageJ. First, the “Analyze” tool was selected and “Set Scale” was conducted according to the scale bar in the image. Then, the whole region of process arbors of each single MBP+ cell was outlined using the “Freehand” tool. The “Measure” tool from “Analyze” was clicked, and the area of each oligodendrocyte (µm^2^) was measured (Figure 4A) [28].

#### 4.7.3. Measurement of Length of Myelinated Axons

The summed length of NF200-labeled axons wrapped by MBP+ oligodendrocyte process was measured in higher magnification images (20×) with ImageJ. The “Freehand line” tool was selected. All MBP+/NF200+ axons (yellow-colored) in the images were drawn and digitized by clicking “Measure” from “Analyze”. The summed length of these axons (µm) in each oligodendrocyte was measured, and the average number from each coverslip was calculated in both the control and isoflurane exposure groups (Figure 4B) [28].

### 4.8. Statistics

Statistical analyses were performed using the GraphPad Prism 8 software. The sample size was determined based on prior experience with this experimental design. Data from each coverslip represent a replicant (n). In each staining, six coverslips with immuno-labeled cells from each group (ISO vs. CON) were randomly selected from three independent batches of cultures (2 from each) for statistical analysis (*n* = 6). The sample sizes are reported on a per-group basis. Comparisons between the control and isoflurane exposure groups were analyzed using the two-tailed Mann–Whitney U test (nonparametric). Significance was defined as *p* < 0.05. Data are presented as mean ± standard deviation (SD) [28].

## 5. Conclusions

In summary, we conclude that early exposure to isoflurane, a frequently used general anesthetic reagent, has disruptive effects on oligodendrocyte development, which includes an increase in oligodendrocyte progenitor cell (OPC) apoptosis, a decrease in OPC proliferation, and the impediment of oligodendrocyte lineage differentiation. Isoflurane exposure also inhibits oligodendrocyte maturation and myelination in primary cultures.

## Data Availability

The datasets generated during the current study are available from the corresponding author upon reasonable request.

## Abbreviations

GA	General anesthetic
OPC	Oligodendrocyte progenitor cell
ISO	Isoflurane
CON	Control
DIV	Day in vitro
ICC	Immunocytochemistry
PBST	Phosphate-buffered saline with triton
PDGFRα	Platelet-derived growth factor receptor alpha
GFAP	Glial fibrillary acidic protein
NeuN	Neuronal nuclei
TUNEL	Terminal deoxynucleotidyl transferase dUTP nick end labeling
EdU	5-ethynyl-2′-deoxyuridine
APC	Adenomatous polyposis coli
MBP	Myelin basic protein
NF200	Neurofilament 200
HBSS	Hank’s balanced salt solution
BSA	Bovine serum albumin
EDTA	Ethylenediaminetetraacetic acid
DMEM	Dulbecco’s modified eagle medium
NBM	Neural basal medium
MAC	Minimum alveolar concentration
AMPA	α-amino-3-hydroxy-5-methyl- 4-isoxazolepropionic acid
NMDA	N-methyl-d-aspartate
cAMP	Cyclic adenosine monophosphate
CREB	cAMP response element-binding protein
GABA	γ-aminobutyric acid
BDNF	Brain-derived neurotrophic factor
trkB	Tropomyosin receptor kinase B
MAPK	Mitogen-activated protein kinase
mTOR	Mammalian target of rapamycin
NGF	Nerve growth factor
NT3	Neurotrophin-3
PFA	Paraformaldehyde
